# Clinical characteristics and physical activity levels of patients with knee osteoarthritis with and without a previous knee injury: A cross-sectional study

**DOI:** 10.1016/j.ocarto.2025.100741

**Published:** 2026-01-05

**Authors:** Richard E. Magony, Jeffrey S. Brooks, Jenna M. Schulz, Derek N. Pamukoff, Jane S. Thornton

**Affiliations:** Western University, London, ON, Canada

**Keywords:** Clinical characteristics, Knee injury, Osteoarthritis

## Abstract

**Objective:**

To compare clinical characteristics and physical activity levels of patients with mild-to-moderate knee osteoarthritis (OA).

**Methods:**

A total of 262 individuals with mild-to-moderate knee OA recruited from the Technology, Exercise Programming, and Activity Prescription for Enhanced Mobility (TEAM) Study (NCT04544904) were categorized into two groups: 1) no previous knee injury (NTOA group), or 2) previous knee injury (PTOA group). We collected participants’ demographics, Knee Injury and Osteoarthritis Outcome Score (KOOS) subscales, Arthritis Self-Efficacy questionnaire, Intermittent and Constant Osteoarthritis Pain (ICOAP) questionnaire, previous sport participation, radiographic OA features, and medical history. A smartphone application measured daily steps and self-reported physical activity levels were measured using the Global Physical Activity Questionnaire. A 30-s chair-stand and 40-m walk test assessed physical function.

**Results:**

The NTOA group was older (62.3 ± 9.0 vs. 58.2 ± 7.4 years, *p* < 0.05) and had lower daily steps (4372 ± 263 vs. 5776 ± 445 steps, *p*_*adj*_ < 0.05) and previous sport participation (62.6 % vs. 81.7 %, *p* < 0.05) than the PTOA group. Anthropometric measurements, sex, radiographic OA scores, comorbidities, smoking, and alcohol consumption were not different. The NTOA group had lower KOOS Pain (58.0 ± 1.2 vs. 64.3 ± 2.1, *p*_*adj*_ < 0.05) and Activities of Daily Living (65.6 ± 1.3 vs. 73.9 ± 2.3, *p*_*adj*_ < 0.05), higher ICOAP (44.2 ± 1.6 vs. 34.7 ± 2.8, *p*_*adj*_ < 0.05), longer 40-m walk times (27.1 ± 0.4 vs. 24.1 ± 0.7 s, *p*_*adj*_ < 0.05) and lower 30-s chair-stand repetitions (12.1 ± 0.4 vs. 14.3 ± 0.7, *p*_*adj*_ < 0.05).

**Conclusion:**

The PTOA group had higher daily steps and better physical and psychological health status than the NTOA group, independent of age and sex. Physical activity interventions may benefit those with NTOA more given their lower activity and greater symptomatology.

**Significance and Innovations**

• Individuals with knee OA and previous PTOA-associated injuries had higher physical function, better physical and psychological patient-reported health outcomes, and higher daily steps compared to those without previous PTOA-associated knee injuries. These differences were independent of the groups' age differences, suggesting that individuals with NTOA and PTOA have unique clinical characteristics and may benefit from different treatment strategies.
• There were no differences in radiographic OA features between individuals with and without previous PTOA-associated injuries, demonstrating a discrepancy between radiological and clinical findings that further supports the prioritization of symptomatology in clinical management and inclusion criteria for PTOA cohorts in research.

## 1 Introduction

Osteoarthritis (OA) is the most common chronic joint disease worldwide and is the leading contributor to disability in Canada, affecting over 4.4 million Canadians [1]. Given the rising global health burden of OA, preventive strategies and therapeutic interventions will be critical to alleviate suffering in the large population of individuals with OA [2]. Osteoarthritis associated with prior trauma or injury is referred to as post-traumatic OA (PTOA) and contributes up to 12 % of all symptomatic OA cases [3]. Compared with other joints, PTOA most often affects the knee and is associated with anterior cruciate ligament (ACL), collateral ligament, meniscal, chondral, and multi-structure injuries, as well as intra-articular fractures and dislocations [4]. Traumatic knee injuries most commonly affect younger adults largely due to higher engagement in physical activity including sport, which can lead to earlier development of OA signs and symptoms compared to OA resulting from non-traumatic causes [[5], [6], [7]].

There is some evidence that clinical characteristics of individuals with knee PTOA may be distinct from individuals with non-traumatic OA (NTOA) and warrant unique treatment strategies. However, PTOA group definitions are inconsistent across studies and make it difficult to determine if their findings truly represent a unique clinical profile of individuals with knee PTOA [[6], [7], [8]]. One study comparing individuals with PTOA or primary OA undergoing knee arthroplasty found that the PTOA group was younger, more often male, and had fewer comorbidities and lower anticoagulant usage compared to the primary OA group, but it included individuals with radiographic OA only [7]. Including individuals with clinical OA is important as symptomology is a key component of global OA definitions and significantly impacts health-related quality of life in those living with OA [9,10]. Another study comparing individuals with PTOA to those with primary OA also identified age and sex differences, but the PTOA group reported higher alcohol consumption and risk of osteoporotic fractures and psychosis but lower risk of diabetes, hypertension and hypothyroidism compared to the primary OA group [6]. However, OA diagnosis (radiographic or clinical) was not specified and individuals with either knee or hip PTOA were included, which may result in different clinical profiles due to their distinct pathophysiology and clinical presentation [6,11]. Finally, a study comparing individuals with knee OA with or without a prior knee injury also identified age and sex differences [8]. Those with previous knee injury had higher 30-s chair-stand test scores, higher physical activity levels, and lower body mass index (BMI). However, they also experienced longer knee pain symptom duration, more painful body sites, and lower quality of life [8]. Additionally, this study did not distinguish knee injury type or severity, which likely led to inclusion of individuals with injuries unrelated to PTOA [8]. Therefore, to ascertain whether PTOA represents a meaningfully different phenotype of knee OA than NTOA, and to determine if individuals with knee PTOA may benefit from tailored clinical management, further descriptive studies with robust selection criteria of knee PTOA cohorts are necessary.

Therefore, the primary aim of this study was to compare clinical characteristics between patients with mild-to-moderate knee OA with and without previous knee injuries associated with PTOA. Patients were recruited from a clinical trial (NCT04544904) and may not have reflected a representative sample of the general public with OA. We hypothesized that the PTOA group would be younger and have higher physical activity levels, lower BMI, lower waist circumference, faster 40-m fast-paced walk speed, higher 30-s chair-stand test scores, and fewer comorbidities compared to the NTOA group. Data will add to a growing body of evidence suggesting that unique OA phenotypes may benefit from individualized treatment strategies to optimize patient outcomes [12].

## 2 Patients and methods

### 2.1 Design

This was a descriptive, cross-sectional study on participants’ characteristics in their baseline assessment in the Technology, Exercise Programming, and Activity Prescription for Enhanced Mobility (TEAM) Study (ClinicalTrials.gov: NCT04544904), an unblinded randomized controlled trial approved by the Lawson Health Research Institute (ID #10428) and the University of Western Ontario Health Science Research Ethics Board (ID #116604) [13]. The overall objective of the study was to determine the effectiveness of physical activity prescription on patient-reported outcomes, physical activity levels, and functional outcomes in individuals with mild-to-moderate hip or knee OA. Methods were reported following the Strengthening the Reporting of Observational Studies in Epidemiology (STROBE) guidelines [14].

Our study's inclusion criteria were the same as in the TEAM Study clinical trial, except only those with knee OA were included [13]. Participants were at least 40 years of age, had mild-to-moderate knee OA based on clinical or radiographic criteria as diagnosed by a physician [15], and had access to a smartphone with internet access. Participants with severe radiographic knee OA were included if they received a clinical diagnosis of moderate knee OA. Based on the American College of Rheumatology guidelines, a clinical diagnosis of OA was made when participants met three out of the following six criteria: age >50 years, morning stiffness <30 min in duration, crepitus, bony tenderness, bony enlargement, and no palpable warmth [15]. Musculoskeletal radiologists assessed anteroposterior knee radiographs and their diagnoses were converted to Kellgren and Lawrence (KL) grades [16]. Individuals were excluded from the study if they [1]: were deemed a surgical candidate for joint replacement surgery within one year or within the study period by an orthopedic surgeon [2]; had inflammatory arthritis (rheumatoid, psoriatic, or disease-modifying anti-rheumatic drug exposure) [3]; had unstable or uncontrolled medical conditions that precluded physical activity prescription [4]; were unable or unwilling to complete follow-up during the study period; or [5] could not communicate in English. In total, 1503 participants were screened and 872 were deemed eligible for the TEAM Study. Out of eligible participants, 456 declined study participation and 60 were lost to follow-up. A total of 356 participants completed their baseline assessment, and 94 were excluded from this cross-sectional study for having hip OA only. Informed consent and data were collected using a secure web-based software program (REDCap, Vanderbilt University). Participants completed patient-reported outcomes online using a unique REDCap link sent via email, and all other outcome measures were entered into REDCap by a member of the research team. The research team staff conducted recruitment and enrolment for all participants.

### 2.2 Clinical and radiographic characteristics

Information was collected on participants’ demographics, medical history, medication use, smoking status, alcohol consumption, history of injury/trauma/surgery, and family history of OA via completion of a Demographics and Medical History questionnaire prior to or during the baseline assessment. All participants underwent weightbearing anteroposterior knee radiographs, which were assessed by musculoskeletal radiologists using the KL system and used to report on structural features of OA in this cohort [16]. Participants were categorized dichotomously based on whether they had a history of ACL, collateral ligament, meniscal, chondral, or multi-structure knee injuries, or intra-articular fractures or dislocations (PTOA group), versus those without such injuries (NTOA group). A physical assessment measured height (cm), mass (kg), BMI (kg/m^2^), and waist circumference (cm). A 30-s chair-stand test and a 40-m fast-paced walk test assessed lower extremity strength and walking capability, respectively.

### 2.3 Patient reported outcomes

Several patient-reported questionnaires assessed levels of function, pain, symptoms, mental health, and fatigue to evaluate symptomatic knee OA severity. The Knee Injury and Osteoarthritis Outcome Score (KOOS) consists of 42 items across five subscales: KOOS Symptoms (KOOS_S_), KOOS Pain (KOOS_P_), KOOS Activities of Daily Living (KOOS_ADL_), KOOS Sport and Recreation Function (KOOS_SR_), and KOOS Quality of Life (KOOS_QoL_). All questions are scored on a 5-point Likert scale (0–4) and converted to a total score from 0 to 100 with higher scores representing less severe knee problems [17]. The Intermittent and Constant OA Pain (ICOAP) questionnaire consists of 11 items scored on a 5-point Likert scale (0–4) and converted to a total score from 0 to 100 with higher scores representing more extreme pain [18]. The Patient Global Assessment of Health Status (PGAHS) records a patient's self-reported assessment of their overall health status from 0 to 100, with higher scores representing better overall health [19]. The Patient Acceptable Symptom State (PASS) asks a single question regarding the patient's satisfaction with their current health state [20]. The Arthritis Self-Efficacy (ASE) questionnaire consists of 8 items scored from 0 to 10, converted to a total score from 0 to 100 with higher scores representing greater knee-related self-efficacy [21]. The Center for the Epidemiological Studies - Depression Scale (CESD-R) consists of 20 items scored on a 5-point Likert scale (0–4) and converted to a total score from 0 to 80 with higher scores representing a higher risk of clinical depression [22]. Finally, the Visual Analog Scale to Evaluate Fatigue Severity (VAS-F) consists of 18 items scored from 0 to 100 and converted to a total score from 0 to 100 with higher scores representing greater fatigue severity [23].

### 2.4 Physical activity status

We evaluated device-based physical activity levels by measuring daily steps using the Myrecovery smartphone application (myrecovery.ai). The application connected to the participant's smartphone's health application (i.e., Google Fit or Apple Health), which has been validated for step measurement across various walking conditions, comparable to accelerometers [24,25]. The phone's accelerometer recorded step counts, which were stored in the phone's health application and extracted by the Myrecovery application [26]. Average daily step count data from the week of participants' assessment was extracted from the application. Self-reported physical activity levels were evaluated using the Global Physical Activity Questionnaire (GPAQ). The GPAQ is a 22-item questionnaire designed to estimate participants' physical activity levels in work, transport, and leisure time in minutes of moderate to vigorous physical activity (MVPA). The total GPAQ score was calculated by summing MVPA minutes from each section, with total metabolic equivalent of task (MET)-min per week determined by multiplying total physical activity minutes by intensity-specific MET factors (4 for moderate, 8 for vigorous) [27].

### 2.5 Statistical analysis

All data analyses were performed by two research team members (RM and JB) using SPSS version 29.0 (IBM Corp., Armonk, NY). Data normality was assessed using the Shapiro-Wilk test, and outliers were visually inspected using boxplots. Descriptive statistics were presented as means with standard deviations for parametric continuous data and medians with interquartile ranges for non-parametric continuous data, or numbers of cases with frequencies for categorical data. Independent samples t-tests and Mann-Whitney U tests were performed to compare parametric and non-parametric continuous outcomes, respectively, between the NTOA and PTOA groups. Adjusted analyses were performed using linear mixed effects models with age and sex entered as covariates given their known differences between NTOA and PTOA groups and their effects on OA pathophysiology and clinical presentation [[6], [7], [8],^28^,^29^]. Group membership was entered as a fixed effect and participants were entered as a random effect. Separate models were created for height, weight, waist circumference, BMI, 40-m fast-paced walk time, 30-s chair-stand repetitions, each patient-reported outcome measure score, daily steps, and alcohol consumption level. Categorical outcomes were compared using the χ ^2^ test or Fisher exact test where appropriate. An alpha level was set at p ≤ 0.05 and multiple comparison error correction using the Benjamin-Hochberg procedure was applied to control the False Discovery Rate for all tests [30]. Effect sizes for unadjusted analyses were reported as Cohen's d for parametric continuous variables, rank-biserial correlation (r) for non-parametric continuous variables, and phi coefficient (φ) for categorical variables. In addition, a supplementary analysis was completed by entering characteristics with significant between-group differences into a multivariable logistic regression model to determine which variables were associated with having a previous knee injury (see Supplementary Material 1).

## 3 Results

### 3.1 Clinical and radiographic characteristics

A total of 262 (47 female and 115 male) individuals with mild-to-moderate knee OA participated in this study. The PTOA group consisted of 67 (25.6 %) participants who reported a prior knee injury strongly associated with PTOA, and the remaining 195 (74.4 %) participants were allocated to the NTOA group [4]. Types of knee injuries reported by individuals in the PTOA group were: 16 cruciate ligament injuries, 4 collateral ligament injuries, 30 meniscal tears, 4 fractures, 6 chondral injuries, and 6 dislocations.

Participants in the PTOA group were on average 4.1 years younger than participants in the NTOA group (58.2 ± 7.4 vs. 62.3 ± 9.0 years), had higher daily steps (4947 IQR [2646–8703] vs. 3784 [1769–6276] steps) and a higher rate of previous sport participation (81.7 % vs. 62.6 %, all p < 0.05, Table 1).

**Table 1:** Comorbidities of individuals in the NTOA and PTOA groups, grouped by related conditions/diseases.

	NTOA (n = 195)	PTOA (n = 67)	Effect size (φ)	*P*-value
Cardiovascular diseases	50 (25.6)	12 (17.9)	−0.079	0.199
Blood diseases	20 (10.3)	5 (7.5)	−0.041	0.502
Respiratory diseases	65 (33.3)	21 (31.3)	−0.018	0.765
Metabolic diseases	116 (59.5)	33 (49.3)	−0.090	0.145
Gastrointestinal diseases	59 (30.3)	19 (28.4)	−0.018	0.769
Musculoskeletal conditions	84 (43.1)	23 (34.3)	−0.078	0.209
Cancer	26 (13.3)	4 (6.0)	−0.101	0.102
History of falling	5 (2.6)	1 (1.5)	−0.031	1.000
Hernia	19 (9.7)	6 (9.0)	−0.012	0.850
Liver disease	0 (0)	2 (3.0)	0.150	0.065
Kidney disease	4 (2.1)	0 (0)	−0.073	0.575

Data are reported as number of cases (%).

Participants in the PTOA group performed the 40-m fast-paced walk test an average of 3.3 s faster than participants in the NTOA group (23.2 [20.2–26.7] vs. 26.5 [22.6–30.1]

sec) and they completed two more repetitions during the 30-s chair-stand test on average (13 [11.0–16.75] vs. 11 [8.5–15.0], all p < 0.05, Table 1).

There were no differences in KL radiographic OA grades, height, weight, waist circumference, BMI and proportions of males and females (Table 1) or comorbidities (Table 2) between NTOA and PTOA groups (p > 0.05).

**Table 2:** Clinical characteristics of NTOA and PTOA groups.

	NTOA (n = 195)	PTOA (n = 67)	Effect size	*P*	NTOA (n = 195)	PTOA (n = 67)	*P*
aAge (y)	**62.3**±**9.0**	**58.2**±**7.4**	**d = 0.478**	**0.013**			
Sex							
*Male*	82 (42.1 %)	33 (49.3 %)					
*Female*	113 (57.9 %)	34 (50.7 %)	φ = 0.063	0.463			
Height (cm)	bn = 181 169.0 [161.8–177.8]	bn = 64 170.8 [163.9–179.5]	r = −0.087	0.287	170.1 ± 0.5	170.5 ± 0.9	0.758
Weight (kg)	bn = 181 86.1 [75.3–100.7]	bn = 64 92.5 [82.1–104.7]	r = −0.105	0.186	91.1 ± 1.5	89.6 ± 2.5	0.704
Waist circumference (cm)	bn = 181 104.3 [94.6–114.3]	bn = 64 106.9 [98.4–111.5]	r = −0.051	0.559	106.2 ± 1.2	104.6 ± 2.1	0.731
BMI (kg/m^2^)	bn = 181 30.0 [26.6–34.7]	bn = 64 31.5 [28.0–35.2]	r = −0.071	0.400	31.4 ± 0.5	31.0 ± 0.8	0.729
KL grade	bn = 192	bn = 63					
*<2*	4 (2.1 %)	4 (6.3 %)					
*2*	79 (41.1 %)	20 (31.7 %)					
*3*	86 (44.8 %)	32 (50.8 %)					
*4*	23 (12.0 %)	7 (11.1 %)	φ = 0.129	0.238			
40-m fast-paced walk time (s)	bn = 181	bn = 64	**r = -0.263**	**0.001**	**27.1**±**0.4**	**24.1**±**0.7**	**0.013**
	**26.5 [22.6**–**30.1]**	**23.2 [20.2**–**26.7]**					
30-s chair-stand repetitions	bn = 181 **11 [8.5**–**15.0]**	bn = 64 **13 [11.0**–**16.75]**	**r = -0.197**	**0.020**	**12.1**±**0.4**	**14.3**±**0.7**	**0.031**
KOOS (total)KOOS_QoL_							
KOOS_S_	57.1 [46.4–71.4]	57.1 [46.4–71.4]	r = −0.028	0.696	58.1 ± 1.2	61.8 ± 2.1	0.244
KOOS_P_	58.3 [44.4–72.2]	63.9 [52.8–72.2]	r = −0.118	0.139	**58.0**±**1.2**	**64.3**±**2.1**	**0.032**
KOOS_ADL_	**69.1 [51.5**–**79.4]**	**75.0 [58.8**–**88.2]**	**r = -0.160**	**0.031**	**65.6**±**1.3**	**73.9**±**2.3**	**0.012**
KOOS_SR_	45.0 [25.0–70.0]	45.0 [30.0–65.0]	r = −0.021	0.547	45.6 ± 1.9	47.6 ± 3.2	0.731
KOOS_QoL_	37.5 [18.8–50.0]	37.5 [25.0–50.0]	r = −0.048		35.9 ± 1.4	39.6 ± 2.3	0.317
ICOAP	bn = 193	36.4 [18.2–52.3]	r = −0.121	0.093	**44.2**±**1.6**	**34.7**±**2.8**	**0.017**
	43.2 [22.7–61.4]						
PGAHS	bn = 193 **57.0 [34.0**–**70.0]**	**66.0 [50.0**–**78.0]**	**r = -0.178**	**0.025**	**53.0**±**1.6**	**63.9**±**2.7**	**0.007**
PASS	bn = 193						
Yes	63 (32.6 %)	22 (32.8 %)					
No	130 (67.7 %)	45 (67.2 %)	φ = 0.002	0.977			
ASE	bn = 193 **56.3 [37.5**–**68.8]**	**67.5 [45.0**–**78.8]**	**r = -0.175**	**0.021**	**53.9**±**1.5**	**63.6**±**2.6**	**0.012**
CESD-R	bn = 192 **16.8 [13.8**–**22.2]**	**15.0 [13.2**–**18.6]**	r = −0.116	0.144	**19.5**±**0.5**	**16.9**±**0.9**	**0.038**
VAS-F	bn = 192 **40.0 [27.6**–**50.4]**	**32.3 [23.2**–**48.7]**	r = −0.107	0.164	**39.4**±**1.0**	**34.4**±**1.8**	**0.035**
GPAQ							
*Moderate MET-min/week*	720.0 [180.0–1680.0]	960.0 [360.0–1800.0]	r = −0.084	0.304	1317 ± 143	1405 ± 248	0.762
*Vigorous MET-min/week*	**0.0 [0.0**–**0.0]**	**0.0 [0.0**–**720.0]**	**r = -0.181**	**0.025**	480 ± 134	657 ± 232	0.773
*Total MET-min/week*	880.0 [240.0–1960.0]	1500.0 [400.0–2600.0]	r = −0.124	0.121	1798 ± 220	2061 ± 381	0.726
Daily steps	**3784 [1769–6276]**	**4947 [2646–8703]**	**r = -0.193**	**0.021**	**4372**±**263**	**5776**±**445**	**0.027**
Alcohol consumption (drinks/day)	bn = 155 2.0 [0.0–6.0]	bn = 60 2.0 [0.6–7.8]	r = −0.069	0.429	3.7 ± 0.4	4.6 ± 0.6	0.342
Smoking status	bn = 155	bn = 60					
*Lifetime abstainer*	87 (56.1 %)	39 (65.0 %)					
*Former occasional smoker*	23 (14.9 %)	10 (16.7 %)					
*Former daily smoker*	32 (20.6 %)	8 (13.3 %)					
*Current occasional smoker*	6 (3.9 %)	2 (3.3 %)					
*Current daily smoker*	7 (4.5 %)	1 (1.7 %)	φ = 0.116	0.656			
Family history of OA	bn = 155	bn = 60					
*Yes*	82 (52.9 %)	29 (48.3 %)					
*No*	73 (47.1 %)	31 (51.7 %)	φ = −0.041	0.632			
Previous sport participation	bn = 155	bn = 60					
*Yes*	**97 (62.6 %)**	**49 (81.7 %)**					
*No*	**58 (37.4 %)**	**11 (18.3 %)**	**φ = 0.183**	**0.027**			
Level of sport participation	bn = 94	bn = 47					
*Non-organized sport*	14 (14.9 %)	4 (8.5 %)					
*Recreational sport*	49 (52.1 %)	18 (38.3 %)					
*Competitive sport*	24 (25.5 %)	17 (36.2 %)					
*Varsity sport*	6 (6.4 %)	4 (8.5 %)					
*Elite sport*	1 (1.1 %)	4 (8.5 %)	φ = 0.247	0.146			
Medications							
*Yes*	177 (90.8 %)	59 (88.1 %)					
*No*	18 (9.2 %)	8 (11.9 %)	φ = −0.040	0.627			

An alpha level of 0.05 was established a priori for significance. Significant findings are bolded. Median [inter-quartile range] and mean ± SE are shown for unadjusted and adjusted analyses, respectively, of continuous variables. Number of cases (proportion) is shown for categorical variables.

NTOA, non-traumatic osteoarthritis; PTOA, post-traumatic osteoarthritis; BMI, body mass index; KL, Kellgren-Lawrence; KOOS, Knee Injury and Osteoarthritis Outcome Score; ICOAP, Intermittent and Constant Osteoarthritis Pain; PGAHS, Patient Global Assessment of Health Status; PASS, Patient Acceptable Symptom State; ASE, Arthritis Self-Efficacy; CESD-R, Center for the Epidemiological Studies – Depression Scale; VAS-F, Visual Analog Scale – Fatigue Severity; GPAQ, Global Physical Activity Questionnaire; MET-min, metabolic equivalent of task minutes.

a Age was compared using independent *t*-test since data was parametric. Mean ± SD is shown.

b Numbers of cases analyzed are shown within cells for variables with missing data.

### 3.2 Patient reported outcomes

#### 3.2.1 KOOS

**Fig. 1:**
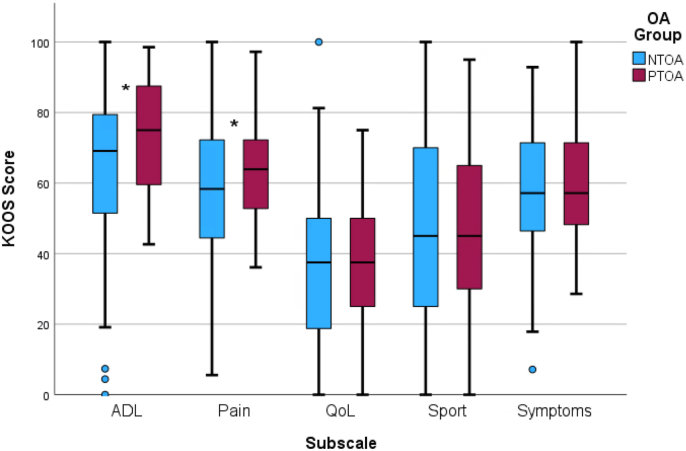
KOOS subscale scores for the NTOA and PTOA groups. Significant differences between groups are marked with asterisks. ADL, Activities of Daily Living; KOOS, Knee Injury and Osteoarthritis Outcome Score; NTOA, non-traumatic osteoarthritis; PTOA, post-traumatic osteoarthritis; QoL, Quality of Life.

There were no significant differences in scores for the KOOS_S_, KOOS_SR_, and KOOS_QoL_ subscales or in proportions reporting “Yes” and “No” in the PASS questionnaire between the NTOA and PTOA groups (Table 1). The PTOA group had higher scores than the NTOA group on the KOOS_P_ after sex adjustment (63.9 [52.8–72.2] vs. 58.3 [44.4–72.2]) and KOOS_ADL_ subscales (75.0 [58.8–88.2] vs. 69.1 [51.5–79.4]), indicating lower pain and higher function during activities of daily living (Fig. 1).

#### 3.2.2 Other questionnaires

The PTOA group scored higher on the ASE questionnaire (67.5 [45.0–78.8] vs. 56.3 [37.5–68.8]) than the NTOA group, demonstrating greater knee-related self-efficacy. The PTOA group had lower ICOAP (36.4 [18.2–52.3] vs. 43.2 [22.7–61.4]) and CESD-R scores (15.0 [13.2–18.6] vs. 16.8 [13.8–22.2]) than the NTOA group after sex adjustment, demonstrating lower pain and depression symptomology. The PTOA group had lower VAS-F scores than the NTOA group (32.3 [23.2–48.7] vs. 40.0 [27.6–50.4]), demonstrating lower fatigue severity. Participants in the PTOA group had higher PGAHS scores (66.0 [50.0–78.0] vs. 57.0 [34.0–70.0]) suggesting their wellness was less impacted by illness or health conditions (all p < 0.05; Table 1).

#### 3.2.3 Physical activity

The PTOA group had higher self-reported vigorous physical activity levels (0.0 [0.0–720.0] vs. 0.0 [0.0–0.0]) compared to the NTOA group (p < 0.05), but there were no differences after adjusting for age and sex. There were no differences in self-reported moderate or total physical activity levels between the NTOA and PTOA groups (p > 0.05; Table 1). Daily steps were weakly correlated with self-reported moderate (r(218) = 0.166, *p* < 0.05) and total physical activity levels (r(218) = 0.136, *p* < 0.05).

## 4 Discussion

Overall, the PTOA group demonstrated higher physical function and better overall health status than the NTOA group based on physical and psychological health outcomes, suggesting that these OA phenotypes have different clinical characteristics and may benefit from different treatment strategies. The percentage of study participants with a previous knee injury strongly associated with PTOA was 25.6 %, which is higher than the 12 % prevalence previously reported in the literature [6,31]. Furthermore, by including only individuals with mild-to-moderate knee OA and excluding individuals with severe knee OA, we may have inflated our cohort's PTOA prevalence relative to the general knee OA population given our evidence of more severe symptomology in the NTOA group.

Individuals with PTOA were younger than those with NTOA, which is consistent with other studies [[6], [7], [8]]. This may be explained by higher rates of sport participation and injuries among adolescents and young adults, which contribute to PTOA development during early adulthood [5,32]. However, there were no significant differences in sex distribution between the NTOA and PTOA groups, contrasting previous studies that reported a higher proportion of males than females in PTOA cohorts [[6], [7], [8]]. Given that females with OA experience more severe symptoms than males regardless of radiographic grade [28,33], our inclusion of individuals with clinical OA may have resulted in a higher proportion of females with PTOA compared to other studies that only included radiographic OA [7]. Symptoms such as pain, stiffness and swelling are hallmark features of clinical OA that impact quality of life and should be considered when defining PTOA cohorts [34].

Our findings regarding physical function, activity levels, and patient-reported outcomes are somewhat consistent with previous findings. In our study, individuals in the PTOA group performed their 40-m fast-paced walk test 3.3 s faster and completed two more repetitions during their 30-s chair-stand test than the NTOA group, which are equal to or greater than the minimum detectable change (MDC) values reported for these respective tests in the knee OA population [35]. These results partially align with another study that found higher chair-stand test scores in individuals with knee OA with a reported prior knee injury versus those without, but no differences in 40-m fast-paced walk times [8]. However, while our study's PTOA group included individuals with specific traumatic knee injuries associated with PTOA, the other study included individuals with minor knee injuries not associated with PTOA which impacts their ability to establish a true PTOA group and compare their clinical characteristics to NTOA. The PTOA group in our study also had higher KOOS_ADL_ subscale scores by 5.9 points, which is greater than minimum important difference (MID) reported in the literature, demonstrating better physical function compared to the NTOA group [36]. Furthermore, the PTOA group had higher average daily steps, similar to a previously reported association of higher self-reported physical activity level and having a prior knee injury [8]. Adjusted analyses did not affect the interpretation of these findings, indicating that higher functionality and daily steps were independent of the PTOA group's younger age and more likely illustrate unique characteristics of PTOA. However, self-reported physical activity levels based on the GPAQ were not different in adjusted analyses. Therefore, these results may reflect a discordance between self-reported and objectively measured physical activity [37]. Given daily steps' strong associations with higher physical function and lower all-cause mortality [38,39], the PTOA group's higher daily steps likely contribute to better physical health status.

Individuals in the PTOA group reported higher psychological health scores compared to the NTOA group with lower levels of depression and fatigue symptoms in adjusted analyses, and higher levels of knee-related self-efficacy and self-reported health status. Although there were differences in KOOS_P_ subscale and ICOAP scores, they did not exceed the MID and minimum importance change reported in the literature, suggesting that they may not represent meaningful differences in pain levels [36]. Nonetheless, the PTOA group's physical and psychological outcomes, independent of their younger age, demonstrate less severe physical and psychological symptomology as unique clinical characteristics of PTOA compared to NTOA. This adds to the limited evidence of psychological health outcomes from two previous studies, which conversely measured lower knee-related quality of life [8] in the knee injury versus no knee injury groups and a higher prevalence of psychosis [6] in the PTOA versus NTOA groups. However, the higher prevalence of psychosis may be explained by the inclusion of individuals with hip PTOA in the PTOA group [6], as schizophrenia is associated with an increased risk of hip fractures [40,41].

There is abundant evidence of the widespread health benefits of physical activity, which improves physical function, alleviates pain, and increases quality of life in individuals with knee OA [42,43]. Therefore, the PTOA group's better physical and psychological health outcomes may be related to its higher inclination towards physical activity reflected by their higher daily steps and rate of previous sports participation [42]. Previous studies have reported fear of pain, physical limitations, lack of positive physical activity experiences, lack of support from healthcare professionals, and negative social comparisons as barriers to physical activity in the knee and hip OA population [44]. Since individuals with PTOA have higher rates of previous sport participation and may engage in higher levels of physical activity, they may have more positive experiences and attitudes toward physical activity, as well as stronger beliefs in its therapeutic efficacy compared to those with NTOA. Thus, compared to individuals with PTOA, those with NTOA may derive greater benefits from tailored physical activity interventions and ongoing support from healthcare professionals with a focus on positive beliefs regarding knee-related self-efficacy and the importance of physical activity [45].

There were no differences in radiographic scores between groups despite the observed differences in pain, function and symptoms. These findings provide further evidence of a discrepancy between radiographic and clinical OA and suggest that clinical decision-making should consider both radiological and clinical findings, as severe symptomology may be unaccompanied by evidence of structural OA [46]. However, individuals with PTOA were younger, and their similar radiographic scores to the NTOA group may indicate that they are reaching certain stages of structural OA development at earlier ages compared to those with NTOA. This is also suggested by the previous finding that individuals with PTOA required total knee arthroplasty at a younger age compared to those with primary OA [7]. Prospective studies with imaging at various timepoints are necessary to clarify if structural disease progression differs between PTOA and NTOA and influences long-term health outcomes. Furthermore, radiographic scores did not differ despite the PTOA group's higher daily steps, suggesting that higher physical activity levels may not be related to the structural progression of knee OA [42]. Regardless, striving to meet general physical activity guidelines in ways that are safe and feasible is an appropriate goal for individuals with NTOA or PTOA and should be recommended in clinical practice [47,48].

Several limitations should be considered when interpreting our results. Firstly, we could not distinguish the precise timing of each participant's reported injury relative to their knee OA onset in all cases. This may have led to an overestimated prevalence of PTOA cases since some individuals categorized in the PTOA group may have experienced injury after knee OA onset. In addition, since the TEAM Study specifically included individuals with mild-to-moderate knee OA only, our conclusions cannot be applied to individuals with severe knee OA. Furthermore, this was an ancillary study within the TEAM Study randomized controlled trial and an a priori power analysis was not conducted for its aims. While we aimed to address confounders in our statistical analyses, participants in the TEAM Study randomized controlled trial were permitted to continue OA treatments such as medications, and physiotherapy, which may have influenced their baseline scores. We were also unable to adequately address psychological factors as potential confounders for physical activity and function, such as fear avoidance and self-efficacy. Physical activity levels were measured using the MyRecovery smartphone application which tracks daily steps taken when the smartphone is carried by the participant [26], but participants may not have always had their smartphone with them throughout the day. Younger participants may carry their phone with them more, which may have contributed to the PTOA group's higher daily steps. The application also does not measure non-ambulatory aerobic activities such as cycling, swimming, or using the elliptical. Thus, it likely underestimated daily steps and overall physical activity levels, and it is unclear if groups were equally affected by underestimation. In addition, self-reported physical activity levels were measured using the GPAQ which has shown inconsistent concurrent validity with device-based measures [49]. Self-reported physical activity levels are often over-estimated in the adult OA population [37]. Therefore, we included both device-based and self-reported physical activity measures to account for their respective limitations [37,50]. Given daily steps were weakly correlated with moderate and total self-reported physical activity levels, the two methods seemed to demonstrate higher agreement when capturing low-to moderate-intensity versus vigorous-intensity physical activity, which is consistent with previous studies [37,51]. However, the Myrecovery application only measured daily steps which does not provide enough information to determine the activity's intensity. Future studies using accelerometry may need to consider measuring additional variables, such as counts per minute, to distinguish between light-, moderate- and vigorous-intensity activities [52].

## 5 Conclusion

Individuals with knee OA and previous PTOA-associated injuries had higher physical function, better physical and psychological patient-reported health outcomes, and higher daily steps than those without previous PTOA-associated knee injuries. These findings were independent of the groups’ age differences, suggesting that individuals with NTOA and PTOA have unique clinical characteristics and may benefit from different treatment strategies. Physical activity interventions, while beneficial for both populations, may confer greater benefits for those with NTOA given their lower activity levels and greater symptom burden. Furthermore, radiographic OA features did not differ between individuals with and without previous PTOA-associated injuries, demonstrating a discrepancy between radiological and clinical findings that further supports the prioritization of symptomatology in clinical management and inclusion criteria for PTOA cohorts in research.

## Grants and financial supporters

Canadian MSK Rehab Research Network.

Academic Medical Organization of Southwestern Ontario Innovation Fund.

Western Strategic Support for Tri-Council Success Seed Fund.

Canadian Institutes of Health Research Canada Research Chair (JST).

Lawson Health Research Institute Internal Research Fund.

## Declaration of competing interests

The authors have no conflicts of interest to disclose.
