# Supplementary material for: Clinical characteristics and physical activity levels of patients with knee osteoarthritis with and without a previous knee injury: A cross-sectional study
Source: Osteoarthr Cartil Open. 2026 Jan 5;8(1):100741. doi: 10.1016/j.ocarto.2025.100741 (PMC12819028; doi:10.1016/j.ocarto.2025.100741)
Supplement: Multimedia component 1 [file mmc1.docx]

**Supplemental Material 1:** Multivariable logistic regression model.

After removing redundant variables, baseline clinical characteristics with significant between-group differences were entered in a multivariable logistic regression model to determine which characteristics were associated with having a previous knee injury. Age and sex were entered in block 1 as covariates, and all other variables were entered in block 2 in a forward stepwise (likelihood ratio) fashion. As shown below, the final model included age, 40-metre fast-paced walk time, and daily steps as significant variables explaining 16.0% of variance in self-reported previous knee injury.

**Block 1 variables (enter):** sex and age.

**Block 2 variables (forward: likelihood ratio):** KOOS-P, KOOS-ADL, 40-m fast-paced walk time, 30-s chair stand repetitions, daily steps, and GPAQ vigorous MET-min/wk.

**Omnibus Tests of Model Coefficients**

| Step | | Chi-square | Df | Sig. |
| --- | --- | --- | --- | --- |
| 1 | Step | 10.075 | 1 | 0.002 |
|  | Block | 10.075 | 1 | 0.002 |
|  | Model | 21.478 | 3 | <0.001 |
| 2 | Step | 3.967 | 1 | 0.046 |
|  | Block | 14.042 | 2 | <0.001 |
|  | Model | 25.445 | 4 | <0.001 |

**Model Summary**

| Step | -2 Log likelihood | Cox & Snell R Square | Nagelkerke R Square |
| --- | --- | --- | --- |
| 1 | 231.712 | 0.093 | 0.136 |
| 2 | 227.744 | 0.110 | 0.160 |

**Variables in the Equation**

| Step |  | B | SE | Wald | Df | Sig. |
| --- | --- | --- | --- | --- | --- | --- |
| 1 | Age | -0.045 | 0.019 | 5.375 | 1 | 0.020 |
|  | Sex | 0.240 | 0.342 | 0.495 | 1 | 0.482 |
|  | 40-m fast-paced walk time | -0.104 | 0.036 | 8.222 | 1 | 0.004 |
|  | Constant | 4.153 | 1.371 | 9.178 | 1 | 0.002 |
| 2 | Age | -0.043 | 0.019 | 4.871 | 1 | 0.027 |
|  | Sex | 0.160 | 0.346 | 0.216 | 1 | 0.642 |
|  | 40-m fast-paced walk time | -0.094 | 0.037 | 6.352 | 1 | 0.012 |
|  | Daily steps | 0.000 | 0.000 | 3.940 | 1 | 0.047 |
|  | Constant | 3.355 | 1.436 | 5.460 | 1 | 0.019 |
